# Factors associated with acquired hypothalamic obesity in children with hypothalamic tumors: a comparative single-center study

**DOI:** 10.1007/s12020-026-04665-w

**Published:** 2026-05-28

**Authors:** Fulya METE KALAYCI, Deniz ÖZALP KIZILAY, Eda ATASEVEN, Mehmet KANTAR, Uluç ÖZKIZILTAN, Elif BOLAT, Aslı SUNER KARAKÜLAH, Damla GÖKŞEN, Şükran DARCAN, Samim ÖZEN

**Affiliations:** 1 Department of Pediatric Endocrinology and Diabetes, Ege University, İzmir, Türkiye Turkey; 2 Department of Pediatric Hematology and Oncology, Ege University, İzmir, Türkiye Turkey; 3 Department of Radiology, Ege University, İzmir, Türkiye Turkey; 4 Department of Neurosurgery, Ege University, İzmir, Türkiye Turkey; 5 Department of Biostatistics and Medical Informatics, Ege University, İzmir, Türkiye Turkey

**Keywords:** Obesity, Children, Hypothalamic Masses, Cranial surgery, Setmelanotide

## Abstract

**Purpose:**

Acquired hypothalamic obesity is a complex condition resulting from hypothalamic damage, often due to tumors. However, not all children with hypothalamic masses develop obesity; while hypothalamic involvement is a recognised factor, the impact of specific surgical approaches and concurrent endocrine deficits on diverse tumour cohorts requires further elucidation. To evaluate clinical, radiological, surgical, and endocrine factors associated with obesity in children with hypothalamic tumors.

**Methods:**

This retrospective single-center study included 34 pediatric patients with hypothalamic tumors (median age at diagnosis: 6.0 years; IQR: 6) followed between 1999 and 2020. Patients with conditions or medications causing obesity were excluded. Data on tumor characteristics, treatment modalities, surgical approach, and endocrine abnormalities were collected. Obesity was defined as BMI > +2 SDS, and patients were grouped as obese and non-obese for comparative analysis.

**Results:**

The most frequent tumor types were optic glioma, pilocytic astrocytoma, and craniopharyngioma. Obesity developed in 15 patients (44.1%). The median time to obesity onset was 3.00 months (IQR: 12.50) after diagnosis, with no significant difference in total follow-up duration between obese and non-obese groups (3.00 vs 3.50 years, p=0.471). At diagnosis, the groups differed significantly regarding surgical history, particularly pterional craniotomy (p=0.048), and the presence of adrenal insufficiency (p=0.020) or hypogonadism (p=0.004). However, univariable logistic regression showed that surgical method was not significantly associated with obesity. Obesity was also significantly associated with insulin resistance (p=0.010) and dyslipidemia (p=0.019).

**Conclusion:**

In conclusion, hypothalamic obesity develops rapidly in children with hypothalamic tumors, often within the first few months following diagnosis. While surgical intervention—particularly via pterional craniotomy—showed a significant association with obesity in bivariate analysis, this association was not statistically significant in univariable logistic regression. The presence of obesity is closely linked to metabolic disturbances such as insulin resistance and dyslipidemia, as well as adrenal insufficiency and hypogonadism. These findings emphasize the necessity of early metabolic screening and personalized follow-up strategies immediately after diagnosis for this high-risk population.

## Introduction

The hypothalamus is a neuroendocrine regulatory region that controls energy, temperature, and hunger [1, 2, 3]. Physical damage to the hypothalamus caused by suprasellar brain tumors, trauma, inflammation, and certain hereditary factors, can lead to hypothalamic dysfunction [1, 4, 5]. Damage to the medial hypothalamus, particularly the arcuate nucleus (ARC) and ventromedial nucleus (VMH), can disturb the balance of peripheral satiety and hunger hormones, such as insulin, ghrelin, and leptin, resulting in excessive weight gain which is known as acquired hypothalamic obesity (aHO) [6, 7].

Hypothalamic obesity (HO) is a hallmark of hypothalamic syndrome, which encompasses a wide range of neuroendocrine, metabolic, and psychosocial disturbances resulting from tumor infiltration or treatment-related injury [8]. It is frequently accompanied by multiple pituitary hormone deficiencies and is associated with increased cardiometabolic comorbidities, including features of metabolic syndrome [5, 6]. In patients with hypothalamic masses, hypothalamic obesity presents a significant challenge that adversely affects quality of life. In a survey of 353 patients with childhood hypothalamic dysfunction following brain tumors; 50.7% of patients were obese [1].

Recent international consensus and diagnostic framework proposed by van Santen et al. and Müller et al. emphasize that evidence of hypothalamic damage on MRI is a core element of aHO, regardless of the underlying tumor type. These studies suggest that the degree of neuroendocrine disruption—manifesting as hyperphagia, rapid weight gain, and multiple pituitary hormone deficits—is the common denominator for aHO. However, while these expert guidance documents highlight the impact of surgical damage, detailed data examining the interplay between specific surgical approaches (e.g., pterional craniotomy) and the resulting endocrine-metabolic profile in a diverse, real-world pediatric cohort remain a critical area for further investigation [1, 9].

Although several studies, particularly in craniopharyngioma, have explored obesity outcomes in children with hypothalamic tumors [10, 11, 12], data encompassing diverse tumor types and different surgical strategies are scarce. Furthermore, despite the recent international consensus statement by [9], which standardized diagnostic criteria and prognostic indicators for aHO, there remains a need for comprehensive clinical data reflecting routine practice.

Therefore, the present study aimed to evaluate clinical, surgical, and endocrine characteristics in a cohort of pediatric patients with hypothalamic tumors and to examine factors associated with the presence of obesity. In particular, we sought to explore whether specific surgical techniques and distinct pituitary hormone deficiencies were more frequently observed in patients with obesity, with the goal of improving early recognition of vulnerable patients in clinical practice.

## Methods

**Fig. 1:**
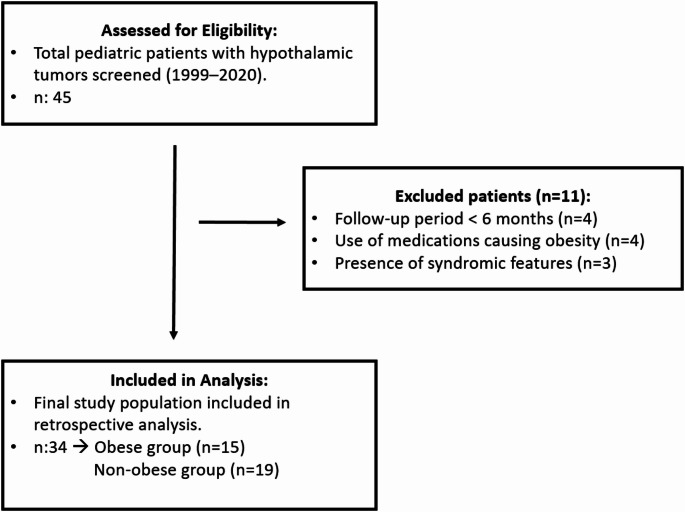
Flow chart of the study population

This study was conducted at the Ege University Faculty of Medicine, Department of Pediatric Endocrinology and Diabetes, a tertiary referral center in İzmir, Türkiye. Data were retrieved from both inpatient records and outpatient clinic follow-up examinations to ensure a comprehensive longitudinal assessment of growth and metabolic parameters. Thirty-four pediatric patients with hypothalamic masses, aged 3 months to 16 years at diagnosis were included. These patients were referred from pediatric oncology and neurosurgery to pediatric endocrinology for follow-up between 1999 and 2020, and patients with at least 6 months of endocrinology follow-up were included because obesity has been reported in the literature to occur 6 months after surgery. As detailed in the flow chart (Fig. 1), a total of 45 patients were screened. Eleven patients were excluded based on the following criteria: a follow-up duration of less than 6 months (*n* = 4), use of medications known to induce weight gain (*n* = 4), and presence of genetic syndromes or other disorders associated with obesity (*n* = 3). The inclusion criteria specifically covered large hypothalamic and pituitary masses that were considered to affect the hypothalamus, either radiologically (e.g. suprasellar extension or involvement of the third ventricular floor or mammillary bodies) or clinically (e.g. panhypopituitarism). The patients with other disorders, syndromes, or those taking medications that could lead to obesity were excluded. The location and size of the mass, type of surgery or radiotherapy, surgical method, presence of calcification, pathology result, presence of hydrocephalus, family history of consanguinity and obesity, anthropometric measurements at diagnosis and end of follow-up, concomitant endocrine pathologies, and comorbidities were retrieved from the files. Body mass index (BMI) was calculated using height and weight [12,13], classifying individuals with a BMI greater than 2 standard deviation scores (SDS) as obese. Due to the retrospective nature of the study, follow-up intervals varied among patients. To ensure data consistency and minimize the impact of missing intermediate data points, BMI SDS at the final follow-up was used as the primary outcome measure for the comparative analysis between groups. Adrenal insufficiency was indicated by basal cortisol levels below 3 µg/dL (83 nmol/L) or a peak response to the low-dose ACTH test < 16 µg/dL (440 nmol/L) [14]. Central hypothyroidism was diagnosed when thyroid-stimulating hormone levels were normal or low and free T4 was below the standard value. Due to the retrospective nature of the study, the specific reference range for the free T4 level was defined as below the age-appropriate lower limit of the reference values provided by the diagnostic kit used at the time of testing. Growth hormone deficiency was diagnosed when insulin-like growth factor-1 levels fell below the age-standardized reference value and clinical symptoms were present. Specifically, diagnosis required both an annual growth velocity of < − 1 SDS for age and IGF-1 levels below − 2 SDS of the age-standardized reference value. Dynamic GH stimulation tests were generally avoided in this cohort, consistent with the principle of not requiring stimulation tests in patients with hypothalamic tumors/intracranial surgery. Hypogonadism was detected by monitoring reproductive hormones in the basal state and during dynamic testing [15]. Evaluation for central hypogonadism and pubertal development was systematically performed at least annually. Dynamic testing (GnRH stimulation test) was performed when basal hormone levels were indeterminate or clinical signs of puberty failed to progress.

In accordance with recently published international expert guidance on acquired hypothalamic obesity (aHO), diagnostic features that characterize aHO were reviewed when interpreting patterns of weight gain in this cohort. The consensus criteria define aHO by three core elements: [1] evidence of hypothalamic damage on MRI secondary to tumor, surgery, irradiation, or another identifiable insult; [2] rapid and clinically meaningful weight acceleration beginning within the first 12 months after the hypothalamic injury, defined in pediatric patients as an increase of ≥ 1.0 BMI SDS that persists for at least 24 months; and [3] the presence of obesity at a level of BMI SDS ≥ + 2.0 [9]. These standardized criteria were not used as inclusion criteria for patient selection in the present study; however, they provide an important clinical framework for interpreting postoperative weight trajectories in children with hypothalamic tumors. Pre- and post-surgical MRI examinations were available and reviewed for all patients, including those with craniopharyngioma, to evaluate the extent of hypothalamic involvement and surgical injury.

Statistical analysis was conducted using IBM SPSS Statistics 24. Normality was assessed using the Shapiro-Wilk test. Continuous variables were presented as mean ± standard deviation for normally distributed data, and as median and interquartile range (IQR) for non-normally distributed data. Independent samples t-test (for normally distributed data) or Mann-Whitney U test (for non-normally distributed data) were used to compare continuous variables. Categorical variables were compared using the Chi-square test or Fisher’s exact test, as appropriate. Univariable logistic regression analysis was performed to identify potential predictors associated with the development of hypothalamic obesity. However, hypogonadism was excluded from this analysis due to the lack of sufficient events (*n* = 1). Furthermore, multivariable logistic regression was not conducted to avoid model overfitting and unstable estimates, given the limited overall sample size and number of obesity events (*n* = 15). Two-tailed P values < 0.05 were considered statistically significant.

The studies involving human participants were reviewed and approved by the Ege University Faculty of Medicine Clinical Research Ethics Committee (Approval No: 24-12.1T/59, approval date: 26 December 2024). Written informed consent was not required for this study in accordance with the national legislation and the institutional requirements.

## Results

**Fig. 2:**
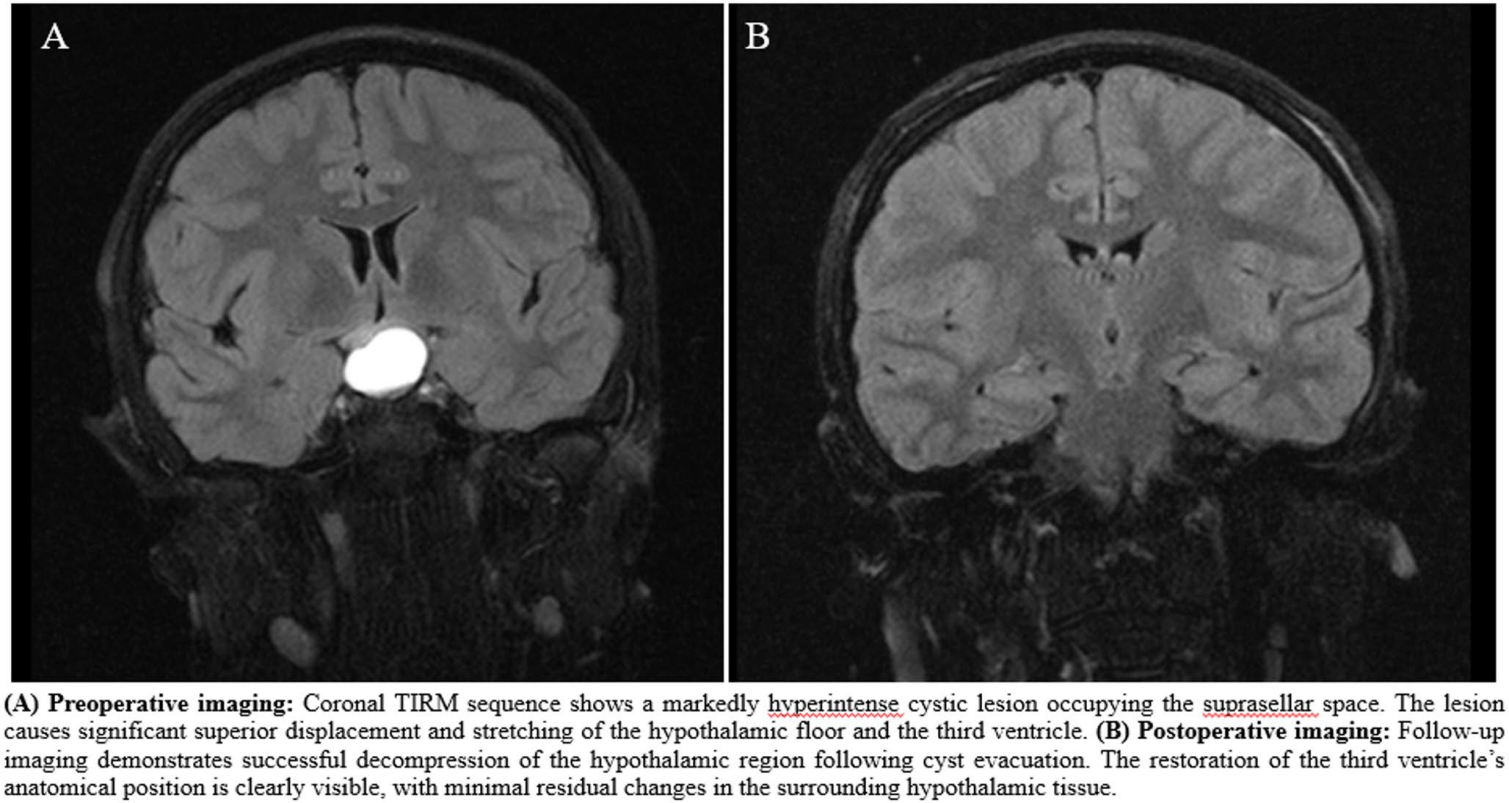
Pre- and postoperative coronal TIRM (dark-fluid) MRI sequences illustrating the extent of the hypothalamic lesion

The study included a total of 34 patients, of which 52.9% were female. The mean long-axis diameter of the masses in the included cases was 33.2 ± 23.04 mm. There were 7 optic gliomas, 8 pilocytic astrocytomas, 7 craniopharyngiomas, 2 pituitary adenomas, 1 chiasmatic glioma, 1 ependymoma, 1 meningioma, 1 glial tumor, 2 germinomas, 2 hypothalamic astrocytoma, 1 pineal tumor, 1 choroid plexus papilloma, and 1 histiocytosis. Radiotherapy was applied to 3 patients, surgery to 14, combined surgery and radiotherapy to 11, and 6 patients were only followed up. Pre- and postoperative MR images demonstrating the extent of the hypothalamic lesion in a representative case are presented in Fig. 2.

At diagnosis, the median age was 6.0 years (IQR: 6.00), while the mean current age was 18.52 ± 7.17 years at the last follow-up. A large majority of the cohort, 32 patients (94.1%), were prepubertal at the time of diagnosis. At the final follow-up, 23 patients (69.7%) still remained prepubertal, indicating a high prevalence of delayed puberty. The median time to obesity onset was 3.00 months (IQR: 12.50) after diagnosis, with no significant difference in total follow-up duration between obese and non-obese groups (3.00 vs. 3.50 years, *p* = 0.471). At the time of diagnosis, 4 out of 34 patients (11.8%) were already obese. During the follow-up period, an additional 11 patients developed obesity, resulting in a total of 15 patients (44.1%) classified as obese at the last follow-up. Patients were categorized into obese (*n* = 15) and non-obese (*n* = 19) groups based on their BMI SDS. Table 1 shows the patients’ anthropometric measurements according to groups.

**Table 1:** Characteristics of patients with and without presenting obesity in terms of anthropometric measurements, treatment modality, accompanying endocrine pathologies and comorbidities at follow up

		Obesity (+) (*n* = 15)	Obesity (-) (*n* = 19)	*p*
**AT DIAGNOSIS**				
Age, year		8.43 ± 4.80	5.08 ± 3.94	**0.033**
Height SDS		-1.19 (IQR: 3.66)	0.34 (IQR: 1.44)	**0.042**
BMI SDS		1.80 (IQR: 3.03)	0.28 (IQR: 1.44)	0.095
Treatment (n, %)	Surgery -	1 (12.5%)	8 (87.5%)	
	Surgery +	14 (56%)	11 (44%)	**0.046**
Surgical method (n, %)	Bifrontal Approach	3 (60%)	2 (40%)	1.000
	Interhemispheric Approach	2 (50%)	2 (50%)	1.000
	Suboccipital Approach	0	2 (100%)	0.142
	Transnasal Approach	2 (50%)	2 (50%)	1.000
	Pterional Craniotomy	6 (100%)	0	**0.048**
	Transcallosal Approach	1 (50%)	1 (50%)	1.000
Years of follow up		3.00 (IQR: 8.00)	3.50 (IQR: 5.02)	0.471
Age, year		21.96 (IQR: 9.67)	13.00 (IQR: 8.78)	**0.018**
BMI SDS		2.75 ± 0.99	0.33 ± 1.00	**< 0.001**
Height SDS		-1.41 ± 2.01	-0.30 ± 0.79	0.058
**Endocrine Pathologies**				
Adrenal insufficiency		9 (69.2%)	4 (30.8%)	**0.020**
Growth hormone deficiency		7 (63.6%)	4 (36.4%)	0.151
Hypogonadism		8 (88.9%)	1 (11.1%)	**0.004**
Diabetes Insipidus		8 (57.1%)	6 (42.9%)	0.201
Hypothyroidism		8 (50%)	8 (50%)	0.515
Multiple hormone deficiency		7 (46.7%)	6 (31.6%)	0.369
**Endocrine Comorbidities**				
Dyslipidemia		10 (66.7%)	5 (33.3%)	**0.019**
Insulin resistance		9 (75%)	3 (25%)	**0.010**
Hepatosteatosis		3 (100%)	0	0.083
Hypertension		1 (50%)	1 (50%)	1.000
Dysglycemia		3 (100%)	0	0.076
Type 2 Diabetes Mellitus		1 (100%)	0	0.441

Continuous variables are presented as mean ± SD or median (IQR), as appropriate. BMI: body mass index, SDS: standard deviation score

No link was found between the amount of weight gain and location of the mass, history of radiation, surgical approach, pre- and post-operative mass size, pathological findings, hydrocephalus, having a family history of obesity. Patients who had surgery were more likely to become obese than those who did not (56% vs. 44%) (*p* = 0.046). The surgical approaches utilized were as follows: 21.7% bifrontal, 17.3% interhemispheric, 8.6% suboccipital, 17.3% transnasal, 26% pterional craniotomy, and 8.6% transcallosal. In the initial bivariate analysis, obesity was significantly more prevalent in patients who underwent pterional craniotomy (*p* = 0.017, Fisher’s exact test). Specifically, among the 6 patients who underwent pterional craniotomy, 3 had craniopharyngioma, 2 had pilocytic astrocytoma, and 1 had meningioma. However, when analyzed via univariable logistic regression, surgical approach—specifically pterional craniotomy—was not found to be a significant independent predictor of hypothalamic obesity (*p* = 0.999). This suggests that while surgical approach may be associated with obesity, its association could not be reliably estimated due to complete separation. Hyperphagia, as assessed through caregiver-reported symptoms, was present in 66.7% of the obese patients. Table 1 reveals endocrine diseases other than obesity and comorbidities related to obesity in this patient population. Of the 11 (32.3%) patients with growth hormone deficiency, only two received recombinant human growth hormone therapy. Hypogonadism was diagnosed in 9 (26.4%) patients (8 in the obese group, 1 in the non-obese group). Hormone replacement therapy was initiated in six of these individuals, all of whom belonged to the obese group. The mean age at which hormone replacement therapy was initiated in these 6 (66.6%) patients was 15.51 ± 2.95 years. One non-obese patient with hypogonadism was not treated. A total of 13 patients (38.2% of the cohort) presented with multiple hormone deficiencies (MHD). While MHD was more frequently observed in the obese group (7 patients, 46.7%) compared to the non-obese group (6 patients, 31.6%), this difference was not statistically significant (*p* = 0.484). The high incidence of MHD, regardless of obesity status, underscores the significant hypothalamic-pituitary damage in this entire cohort. Obesity was more prevalent in cases with adrenal insufficiency (*p* = 0.020) and hypogonadism (*p* = 0.004). As expected, obesity was significantly linked with insulin resistance (*p* = 0.010) and dyslipidemia (*p* = 0.019). Univariable logistic regression analysis identified several variables significantly associated with obesity (Table 2). Among endocrine pathologies, hypogonadism demonstrated the highest odds ratio in univariable analysis (OR = 20.57, 95% CI: 2.15–196.10, *p* = 0.009), followed by adrenal insufficiency (OR = 5.62, 95% CI: 1.24–25.49, *p* = 0.025). Regarding metabolic comorbidities, insulin resistance was associated with 7.5-fold higher odds of obesity (OR = 7.50, 95% CI: 1.49–37.65, *p* = 0.014), while dyslipidemia was also associated with higher odds (OR = 5.60, 95% CI: 1.27–24.64, *p* = 0.023). Additionally, age at diagnosis (OR = 1.19, *p* = 0.044) and current BMI SDS (OR = 8.82, *p* = 0.007) were found to be associated with higher odds of obesity.

**Table 2:** Univariable Logistic Regression Analysis of Factors Associated with Hypothalamic Obesity

	*p*	OR	95% CI	
			Lower	Upper
Age at diagnosis (years)	0.044	1.19	1.00	1.42
Height SDS at diagnosis	**0.044**	0.47	0.22	0.98
Any surgical intervention	0.056	8.90	0.94	83.61
Pterional Craniotomy	0.999	Not estimable*		
Age at last follow-up (years)	**0.027**	1.13	1.01	1.27
Adrenal insufficiency	**0.025**	5.62	1.24	25.49

*Due to complete separation (no non-obese patients in the pterional group), odds ratio and confidence intervals could not be reliably estimated

Regarding the management of obesity, lifestyle modifications and increased physical activity were recommended to all patients identified as obese. However, due to the retrospective design of the study, standardized data regarding the specific duration, intensity, or frequency of physical exercise were not available. Throughout the follow-up period, no significant weight loss was observed; the majority of patients either continued to gain weight or remained within the obese BMI range. In terms of pharmacological interventions, metformin was the only medication utilized in our cohort, with six patients (17.6%) receiving this treatment due to concurrent insulin resistance. No other anti-obesity medications, such as GLP-1 receptor agonists or setmelanotide, were recorded during the follow-up period.

## Discussion

Acquired hypothalamic obesity results from structural or functional disruption of hypothalamic nuclei involved in energy homeostasis. Key regions implicated include paraventricular nucleus (PVN), ARC and VMH, which integrate peripheral hormonal signals such as leptin, insulin, and ghrelin to regulate appetite, satiety, and energy expenditure. Damage to the ARC—home to anorexigenic pro-opiomelanocortin (POMC) and orexigenic neuropeptide Y/agouti-related peptide (NPY/AgRP) neurons—can impair melanocortin signaling, leading to uncontrolled hyperphagia and reduced energy expenditure. In particular, disruption of the leptin–POMC–MC4R pathway has been highlighted as a central mechanism in both genetic and acquired forms of hypothalamic obesity. Recent insights from Argente et al. emphasize that HO shares common downstream neuroendocrine alterations with monogenic MC4R pathway diseases particularly involving melanocortin tone deficiency and autonomic imbalance. These pathophysiological similarities support the rationale for targeting the melanocortin pathway therapeutically, even in acquired cases of HO, such as through MC4R agonists like setmelanotide [3].

In our cohort, although surgical intervention—particularly pterional craniotomy—showed a significant association with obesity in the initial bivariate analysis, univariable logistic regression demonstrated significant unadjusted associations between obesity and adrenal insufficiency, hypogonadism, and age at diagnosis.

Although in our study tumor type was not associated with obesity, there are some studies on the risk factors or treatment options for hypothalamic obesity, mostly in patients diagnosed with craniopharyngioma [10, 11, 12]. This is an indication that the hypothalamus is more susceptible to mechanical damage rather than the type of tumor. Similarly, Şıklar et al. evaluated pediatric patients with craniopharyngioma and reported that 23% were obese at the time of diagnosis, increasing to 38% after surgery [16]. They emphasized that the degree of hypothalamic involvement prior to surgery was a significant determinant of long-term postoperative outcomes, including persistent obesity. Consistent with their findings, the 44.1% obesity rate observed in our cohort underscores that hypothalamic dysfunction following tumor surgery appears to play a central role in the pathogenesis of acquired hypothalamic obesity in children.

In our study patients gained weight at a median of 3.00 months (IQR: 12.50) after diagnosis which is consistent with the literature. Our findings also align with the recently proposed international consensus criteria for acquired hypothalamic obesity (aHO). According to these guidelines, aHO is characterized by early and rapid postoperative BMI acceleration—defined as an increase of ≥ 1.0 SDS within the first 12 months following hypothalamic injury—followed by persistent obesity (BMI SDS ≥ + 2.0) [9]. In our cohort, the median time to significant weight gain was 3.00 months, which is fully consistent with the expected temporal trajectory described in the consensus document. Laura van Iersel et al. reported that rapid weight gain occurs in the first 6–12 months after initial surgery [6]. Paul Dimitri also noted in his review that rapid weight gain often occurs within the first year after surgery and that surgery increases the prevalence of obesity in this patient group [2]. This correspondence supports the view that the weight patterns observed in our patients represent the classical phenotype of aHO, emphasizing the need for early monitoring and intervention following hypothalamic tumor surgery.

Obesity was more frequently observed in patients who underwent surgery, with a particularly higher prevalence in those who had a pterional craniotomy. Specifically, among the 6 patients who underwent pterional craniotomy, 3 patients had craniopharyngioma, 2 patients had pilocytic astrocytoma, and 1 patient had meningioma. This distribution across varied tumor types suggested that the surgical context, rather than tumor histology alone, might be associated with obesity (*p* = 0.017, Fisher’s exact test). However, logistic regression analysis could not reliably estimate the effect of pterional craniotomy due to complete separation (no non-obese patients in this subgroup), reflecting the limited sample size. Therefore, this finding should be interpreted cautiously and cannot be considered evidence of an independent effect of surgical approach.

To explore potential temporal changes, we compared obesity prevalence across two time periods. Although obesity remained common overall, a non-significant decreasing trend was observed between 1999 and 2009 (58.3%) and 2010–2020 (36.4%, *p* = 0.288). While not statistically significant, this observation may reflect improvements in neurosurgical techniques and increased awareness of hypothalamic preservation. Larger studies are required to determine whether such temporal differences represent true changes in clinical practice.

Previous studies in pediatric craniopharyngioma cohorts have similarly examined the relationship between surgical extent and postoperative obesity. For example, Sarkar et al. reported a higher prevalence of obesity in patients undergoing subtotal resection followed by radiotherapy compared with total or near-total resection [10].These findings raise the question of whether obesity is more closely related to the degree of hypothalamic involvement rather than the surgical technique itself. Consistent with van Schaik et al. and Beckhaus et al., our results support the concept that the extent of hypothalamic damage—irrespective of tumor histology—appears to play a central role in subsequent metabolic outcomes [17, 18]. According to the recent international expert guidance by Müller et al., disruption of the posterior hypothalamic nuclei and the third ventricular floor is a key structural correlate of acquired hypothalamic obesity [9]. Such lesions interfere with central satiety signaling pathways, paralleling mechanisms described in monogenic obesity syndromes.

Importantly, discussions with neurosurgical colleagues suggest that the pterional approach itself does not inherently confer a higher likelihood of hypothalamic injury; rather, anatomical complexity and tumor characteristics may determine the degree of hypothalamic involvement. Supporting this interpretation, Roth et al. demonstrated that obesity was strongly associated with radiological involvement of specific hypothalamic regions, particularly the posterior hypothalamus [19]. In contrast to some previous reports [19, 20], we did not observe significant associations between obesity and hydrocephalus, tumor location, or tumor size, which may reflect the limited statistical power of our cohort.

In our study, endocrine pathologies such as adrenal insufficiency and hypogonadism were more frequently observed among patients with obesity. In univariable logistic regression analysis, hypogonadism was associated with approximately 20-fold higher odds of obesity (OR = 20.57, 95% CI: 2.15–196.10, *p* = 0.009), while adrenal insufficiency was associated with over 5-fold higher odds (OR = 5.62, 95% CI: 1.24–25.49, *p* = 0.025). However, these estimates represent unadjusted associations and should be interpreted cautiously given the limited sample size and wide confidence intervals.

The coexistence of multiple pituitary hormone deficiencies may reflect more extensive hypothalamic–pituitary involvement. This pattern is consistent with previous reports demonstrating a high cumulative incidence of hypothalamic–pituitary deficits in pediatric brain tumor survivors, particularly in those with suprasellar or central tumors [21]. Although multiple hormone deficiency (MHD) was not significantly associated with obesity in our cohort (*p* = 0.484), it was numerically more frequent in the obese group (46.7%) compared with the non-obese group (31.6%). This pattern is consistent with previous literature suggesting that greater structural hypothalamic involvement is linked to more pronounced metabolic disturbances [2, 6].

We noted that four out of 34 patients (11.8%) were obese at the time of tumour diagnosis. Although we retained these patients in the ‘Obesity (+)’ group to preserve statistical power, a detailed analysis of the four cases in question support the hypothesis that their obesity was an early manifestation of hypothalamic damage. All four patients presented with significant endocrine deficiencies that are associated with hypothalamic injury at the time of diagnosis: one had hypothyroidism and hypogonadism; one had hypogonadism only; one had hypothyroidism only; and the fourth had panhypopituitarism. The patient with panhypopituitarism (who was diagnosed with a craniopharyngioma) had a BMI SDS of 2.19 and a height SDS of − 1.76 at diagnosis, and these values improved to 1.66 and − 2.14, respectively, at the final follow-up. These clinical findings strongly suggest that their obesity was a direct consequence of tumor effects rather than a pre-existing condition, thus justifying their inclusion in the acquired hypothalamic obesity cohort.

Furthermore, in our cohort, patients in the obese group were significantly older at diagnosis than those in the non-obese group [median 8.43 years (IQR: 4.80) vs. 5.08 years (IQR: 3.94), *p* = 0.033] (Table 1). The obese group also presented with a significantly lower height SDS at diagnosis compared to non-obese patients (*p* = 0.042). Lower height SDS at diagnosis was associated with higher odds of obesity, suggesting early hypothalamic–pituitary dysfunction. It is notable that the trend for shorter stature continued until the end of the follow-up period (Height SDS: -1.41 ± 2.01 vs. -0.30 ± 0.79, *p* = 0.058). This persistent or worsening short stature in the obese group is clinically significant because low height SDS can mathematically inflate BMI SDS, potentially leading to an overestimation of obesity severity in some patients. However, by analysing specific patient data, we demonstrate that obesity progression is the primary issue: Three short-statured patients at diagnosis (mean initial height SDS of − 3.6 ± 0.32 and mean initial BMI SDS of 1.73 ± 0.88) progressed to a mean final BMI SDS of 2.5 (final height SDS of − 2.9 ± 0.77), demonstrating clear progression to obesity despite their short stature. In contrast, one patient with short stature (initial height SDS − 1.76, BMI SDS 2.19) showed improvement in obesity by the end of the follow-up period (final BMI SDS 1.66). The four patients who were already obese at diagnosis (mean initial BMI SDS 2.39 ± 0.19) maintained high obesity levels (final BMI SDS 2.6 ± 0.17).

Gonadal hormones (estrogen and testosterone) also play a protective role in metabolism. While pubertal onset typically occurs between 8.5 and 13 years in girls and 9–13.5 years in boys [15], a large majority of our cohort (94.1%) was prepubertal at diagnosis, and 69.7% remained prepubertal at the end of follow-up. Consequently, our conclusion regarding the strong link between hypogonadism and obesity must be qualified. This limitation was also acknowledged under Study Limitations. The biochemical diagnosis of central hypogonadism is inherently challenging in prepubertal children due to the physiological suppression of the hypothalamic–pituitary–gonadal axis; therefore, this association is likely underestimated. The high rate of persistent prepubertal status also reflects significant growth and pubertal delay resulting from hypothalamic damage, which further complicates the long-term metabolic outcomes of this cohort. Importantly, only 2 of 11 patients with growth hormone deficiency and 6 of 9 with hypogonadism received replacement therapy, highlighting a significant therapeutic dilemma. The most common reason for withholding growth hormone (GH) therapy in these patients was the presence of residual or recurrent tumor mass. This reflects the complex neuro-oncological management, whereby GH replacement therapy may be postponed or contraindicated due to concerns about stimulating tumor growth.

Unlike some studies [1, 10], we did not find a significant association between diabetes insipidus and obesity. Insulin resistance and hyperinsulinemia were the most common metabolic comorbidities observed among patients with obesity, consistent with previous reports. In univariable logistic regression analysis, insulin resistance was significantly associated with higher odds of obesity (OR = 7.50, 95% CI: 1.49–37.65, *p* = 0.014), and dyslipidemia showed a similar association (OR = 5.60, 95% CI: 1.27–24.64, *p* = 0.023). However, given the cross-sectional and retrospective nature of the data, these findings likely reflect metabolic consequences of obesity rather than independent predictive factors.

Although the incidence of dysglycemia appeared higher among obese patients, no significant association with type 2 diabetes was observed. While clinical guidelines often highlight the association between obesity and hypertension [22], this relationship was not statistically significant in our cohort. Longer follow-up may clarify whether additional metabolic complications emerge over time.

Given the multifactorial etiology of hypothalamic obesity—including hypothalamic dysfunction, hormonal deficiencies, reduced mobility, and behavioral dysregulation—multidisciplinary follow-up is essential. Pediatric endocrinologists, neurosurgeons, dietitians, psychologists, and physiotherapists should collaborate from the time of diagnosis to optimize both metabolic control and quality of life. Risk-adapted, individualized monitoring protocols including early hormonal screening and appetite regulation counseling may mitigate long-term morbidity.

Many medical treatments for hypothalamic obesity, such as metformin, diazoxide, octreotide, GLP-1 analogues, oxytocin, and tesofensine, are documented in the literature but yield limited success [2, 4]. Metformin and lifestyle changes have been shown to stabilize weight gain with fewer side effects [5]. We initiated metformin treatment in six patients with insulin resistance. Advising lifestyle changes when an increase in appetite is noticed may be beneficial, as weight gain is particularly rapid in the first few months after surgery. Bariatric surgery is commonly performed in adults, but there is less experience in the pediatric age group [2, 5]. Particularly in patients with hypothalamic obesity, surgical side effects due to comorbidities such as adrenal insufficiency should be assessed on an individual basis [6].

Acquired hypothalamic obesity may involve disruptions in melanocortin signaling due to hypothalamic damage. In a phase 2 open-label trial, subjects aged 6 to 40 years with a history of obesity and hypothalamic damage were treated with setmelanotide. After 16 weeks, 89% of participants achieved a ≥ 5% reduction in BMI, with sustained effects observed over 12 months in a long-term extension [23]. Significant improvements in hunger scores and BMI Z-scores were also reported. These results highlight the therapeutic potential of MC4R pathway modulation, suggesting that our patient group may be a candidate for this treatment.

### Study limitations

Our study has several limitations that warrant consideration. Given the small number of obesity events, regression estimates may be unstable and should be interpreted with caution. First, the small sample size and the retrospective design resulted in non-standardized follow-up intervals and missing intermediate anthropometric data, which precluded the presentation of detailed weight trajectories across different tumor types. Second, the heterogeneity of the cohort, comprising various tumors such as optic gliomas, craniopharyngiomas, and pituitary adenomas, along with diverse treatment modalities, made it difficult to generalise outcomes, given that prognosis varies considerably among these groups. Furthermore, it was not possible to fully account for confounding factors, such as the specific type or duration of chemotherapy and its potential indirect effects on metabolic health. The lack of standardized data on physical activity levels and dietary adherence also limited our assessment of lifestyle interventions. Additionally, the predominance of prepubertal patients may have hindered the biochemical confirmation of central hypogonadism, potentially leading to an underestimation of its true association with obesity. Although longer follow-up periods are necessary to evaluate long-term effects, the rarity of this patient population and the consistent data collection at a single tertiary centre offer a valuable clinical framework for understanding acquired hypothalamic obesity.

## Conclusion

In conclusion, our findings suggest that hypothalamic obesity in children with hypothalamic tumors tends to develop rapidly, with a median onset of 3.00 months following diagnosis. Although surgical intervention—particularly via pterional craniotomy—was associated with a higher prevalence of obesity in initial comparisons, its effect could not be reliably estimated in regression analysis due to the limited sample size.

In univariable logistic regression analysis, hypogonadism, adrenal insufficiency, and older age at diagnosis were significantly associated with obesity. However, these findings represent unadjusted associations and should be interpreted cautiously given the small cohort and absence of multivariable adjustment.

The observed clustering of obesity with multiple endocrine deficits likely reflects more extensive hypothalamic–pituitary involvement. These results highlight the importance of early metabolic monitoring and careful longitudinal follow-up in this vulnerable population. Larger prospective studies incorporating standardized radiological assessment are needed to better clarify the determinants of obesity in children with hypothalamic tumors.

## Data Availability

The data supporting the findings of this study are not publicly available due to institutional and ethical restrictions involving patient confidentiality. However, the datasets are available from the corresponding author upon reasonable request and with permission from the Ege University Faculty of Medicine Clinical Research Ethics Committee.

## Declarations

### Statements

This research complies with the guidelines for human studies and is conducted ethically following the World Medical Association Declaration of Helsinki.
